# Supplementary material for: Q4ddPCR: a flexible, 4-target assay for high-resolution HIV reservoir profiling
Source: Nat Commun. 2026 Feb 20;17:2975. doi: 10.1038/s41467-026-69413-0 (PMC13035820; doi:10.1038/s41467-026-69413-0)
Supplement: Supplementary file 1 — Supplementary Information [file 41467_2026_69413_MOESM1_ESM.pdf]

Supplementary Material for

Q4ddPCR: A Flexible, 4-Target Assay for High-Resolution HIV Reservoir Profiling

Rachel Scheck[1,2], Mark Melzer[3], Gregory Gladkov[2], Louise Leyre[2], Adam R. Ward[2], Daniel B. Reeves[4], Naomi Perkins[1], T. Thinh Huynh[2], Deborah K. McMahon[5,6], Ronald J. Bosch[7], Bernard J. Macatangay[5,6], Joshua C. Cyktor[5], Joseph J. Eron[8], John W. Mellors[5], Rajesh T. Gandhi[9], Lisa Buchauer[3], R. Brad Jones[2*#] and Christian Gaebler[1*#]

# equal contribution

[1] Laboratory of Translational Immunology of Viral Infections, Department of Infectious Diseases and Critical Care Medicine, Charité-Universitätsmedizin Berlin, and Berlin Institute of Health, Berlin, Germany.

[2] Division of Infectious Diseases, Department of Medicine, Weill Cornell Medicine, New York, NY 10065, USA.

[3] Laboratory of Systems Biology of Infectious Diseases, Charité-Universitätsmedizin, Berlin, Germany.

[4] Vaccine and Infectious Disease Division, Fred Hutchinson Cancer Center, Seattle, WA, USA

[5] Division of Infectious Diseases, University of Pittsburgh, Pittsburgh, Pennsylvania, USA.

[6] Department of Infectious Diseases and Microbiology, University of Pittsburgh School of Public Health, Pittsburgh, Pennsylvania, USA.

[7] Center for Biostatistics in AIDS Research, Harvard TH Chan School of Public Health, Boston, Massachusetts, USA.

[8] Division of Infectious Diseases, University of North Carolina, Chapel Hill, North Carolina, USA.

[9] Infectious Diseases Division, Massachusetts General Hospital, Harvard Medical School, Boston, Massachusetts, USA.

Correspondence:

Christian Gaebler, christian.gaebler@charite.de

R. Brad Jones, rbjones@med.cornell.edu

Supplementary Figures

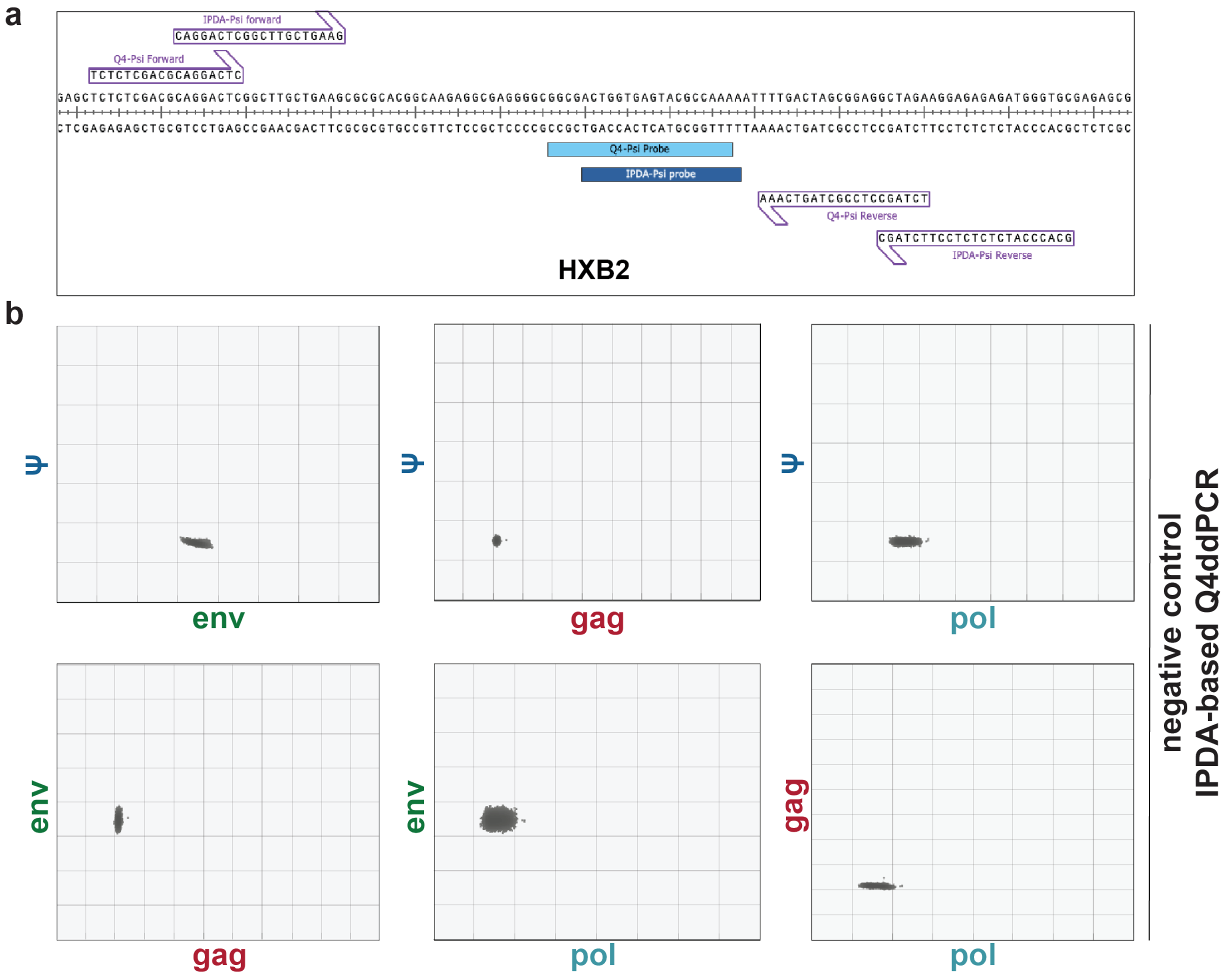

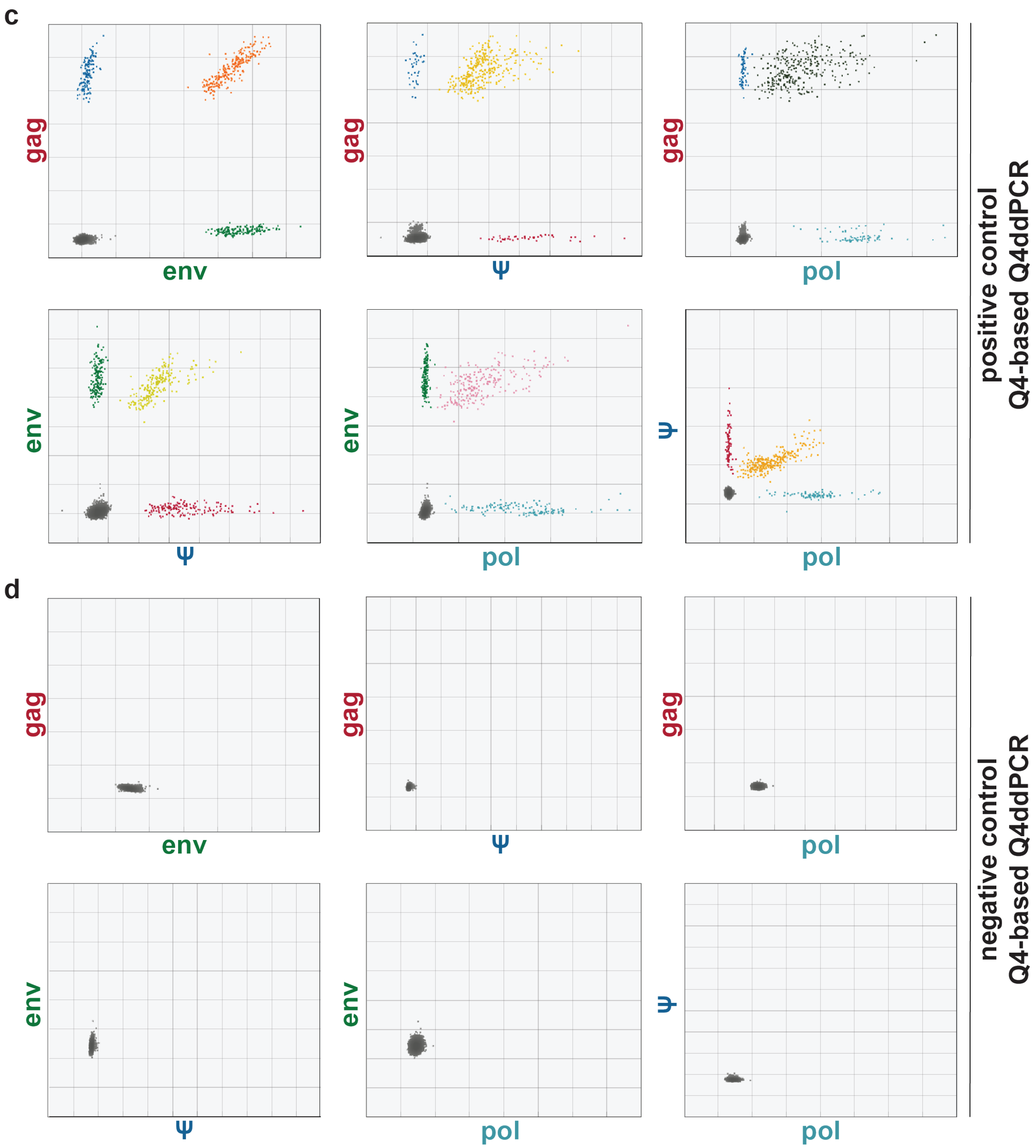
c
gag
env
Ψ
pol
positive control
Q4-based Q4ddPCR
d
negative control
Q4-based Q4ddPCR

Supplementary Figure 1 | *Ψ*-binding site and representative Q4ddPCR controls

a Primer and probe sequences for the Q4- and IPDA-based *Ψ*-targets mapped to the HXB2 reference genome.

b-d Representative 2-dimensional Q4ddPCR plots for IPDA-based Q4ddPCR on negative controls **(b)** and for Q4-based Q4ddPCR on positive **(c)** and negative **(d)** controls. DNA extracted from PBMCs from people without HIV served as negative controls; J-Lat 6.3 DNA served as positive control.

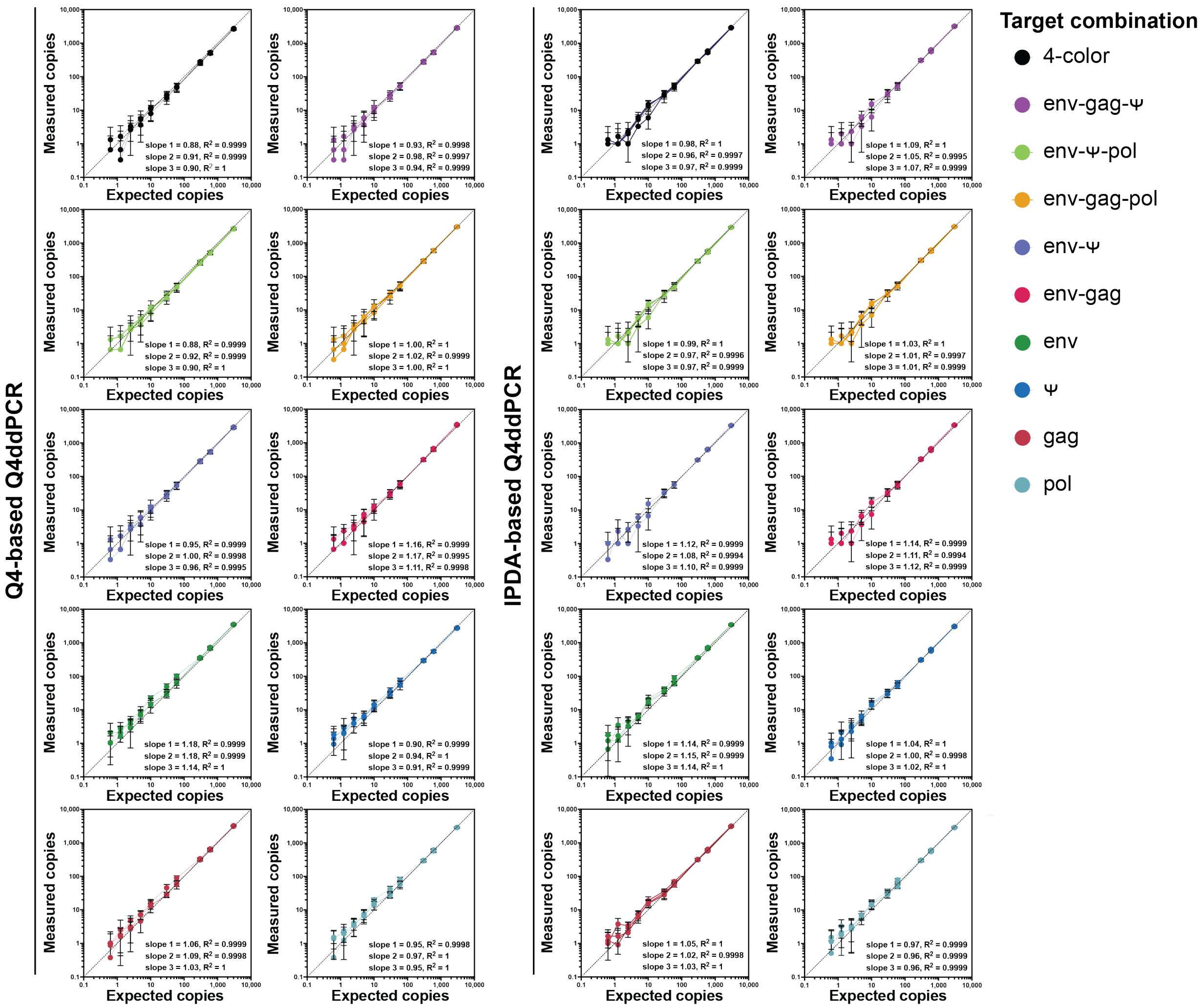

Q4-based Q4ddPCR
IPDA-based Q4ddPCR
Measured copies
Expected copies
slope 1 = 0.88, R² = 0.9999
slope 2 = 0.91, R² = 0.9999
slope 3 = 0.90, R² = 1
slope 1 = 0.93, R² = 0.9998
slope 2 = 0.98, R² = 0.9997
slope 3 = 0.94, R² = 0.9999
slope 1 = 0.98, R² = 1
slope 2 = 0.96, R² = 0.9997
slope 3 = 0.97, R² = 0.9999
slope 1 = 1.09, R² = 1
slope 2 = 1.05, R² = 0.9995
slope 3 = 1.07, R² = 0.9999
slope 1 = 0.88, R² = 0.9999
slope 2 = 0.92, R² = 0.9999
slope 3 = 0.90, R² = 1
slope 1 = 1.00, R² = 1
slope 2 = 1.02, R² = 0.9999
slope 3 = 1.00, R² = 1
slope 1 = 0.99, R² = 1
slope 2 = 0.97, R² = 0.9996
slope 3 = 0.97, R² = 0.9999
slope 1 = 1.03, R² = 1
slope 2 = 1.01, R² = 0.9997
slope 3 = 1.01, R² = 0.9999
slope 1 = 0.95, R² = 0.9999
slope 2 = 1.00, R² = 0.9998
slope 3 = 0.96, R² = 0.9995
slope 1 = 1.16, R² = 0.9999
slope 2 = 1.17, R² = 0.9995
slope 3 = 1.11, R² = 0.9998
slope 1 = 1.12, R² = 0.9999
slope 2 = 1.08, R² = 0.9994
slope 3 = 1.10, R² = 0.9999
slope 1 = 1.14, R² = 0.9999
slope 2 = 1.11, R² = 0.9994
slope 3 = 1.12, R² = 0.9999
slope 1 = 1.18, R² = 0.9999
slope 2 = 1.18, R² = 0.9999
slope 3 = 1.14, R² = 1
slope 1 = 0.90, R² = 0.9999
slope 2 = 0.94, R² = 1
slope 3 = 0.91, R² = 0.9999
slope 1 = 1.14, R² = 0.9999
slope 2 = 1.15, R² = 0.9999
slope 3 = 1.14, R² = 1
slope 1 = 1.04, R² = 1
slope 2 = 1.00, R² = 0.9998
slope 3 = 1.02, R² = 1
slope 1 = 1.06, R² = 0.9999
slope 2 = 1.09, R² = 0.9998
slope 3 = 1.03, R² = 1
slope 1 = 0.95, R² = 0.9998
slope 2 = 0.97, R² = 1
slope 3 = 0.95, R² = 1
slope 1 = 1.05, R² = 1
slope 2 = 1.02, R² = 0.9998
slope 3 = 1.03, R² = 1
slope 1 = 0.97, R² = 0.9999
slope 2 = 0.96, R² = 0.9999
slope 3 = 0.96, R² = 0.9999
Target combination
4-color
env-gag-Ψ
env-Ψ-pol
env-gag-pol
env-Ψ
env-gag
env
Ψ
gag
pol

Supplementary Figure 2 | Linear dynamic range and linearity assessment of Q4ddPCR

J-Lat 6.3 cells, each containing a single intact provirus, were spiked into PBMCs from people without HIV (PWoH). DNA was extracted, and three independent 10-step dilution series (0.625 - 3000 copies) were generated and measured using both Q4-based and IPDA-based Q4ddPCR. Expected copy numbers (x-axis) were compared with measured copies (y-axis) for target combinations used to infer intactness in Q4ddPCR as well as for individual targets (*env, Ψ, gag, pol*). Copy numbers for 2-, 3-, and 4-target combinations were shear-corrected using the RPP30 assay. Each dilution series included eight replicate wells. Target combinations are color-coded; mean values and standard deviations are shown. Slopes and R^2 values derived from linear regression are indicated for each dilution curve (1 - 3). Source data are provided as a Source Data file.

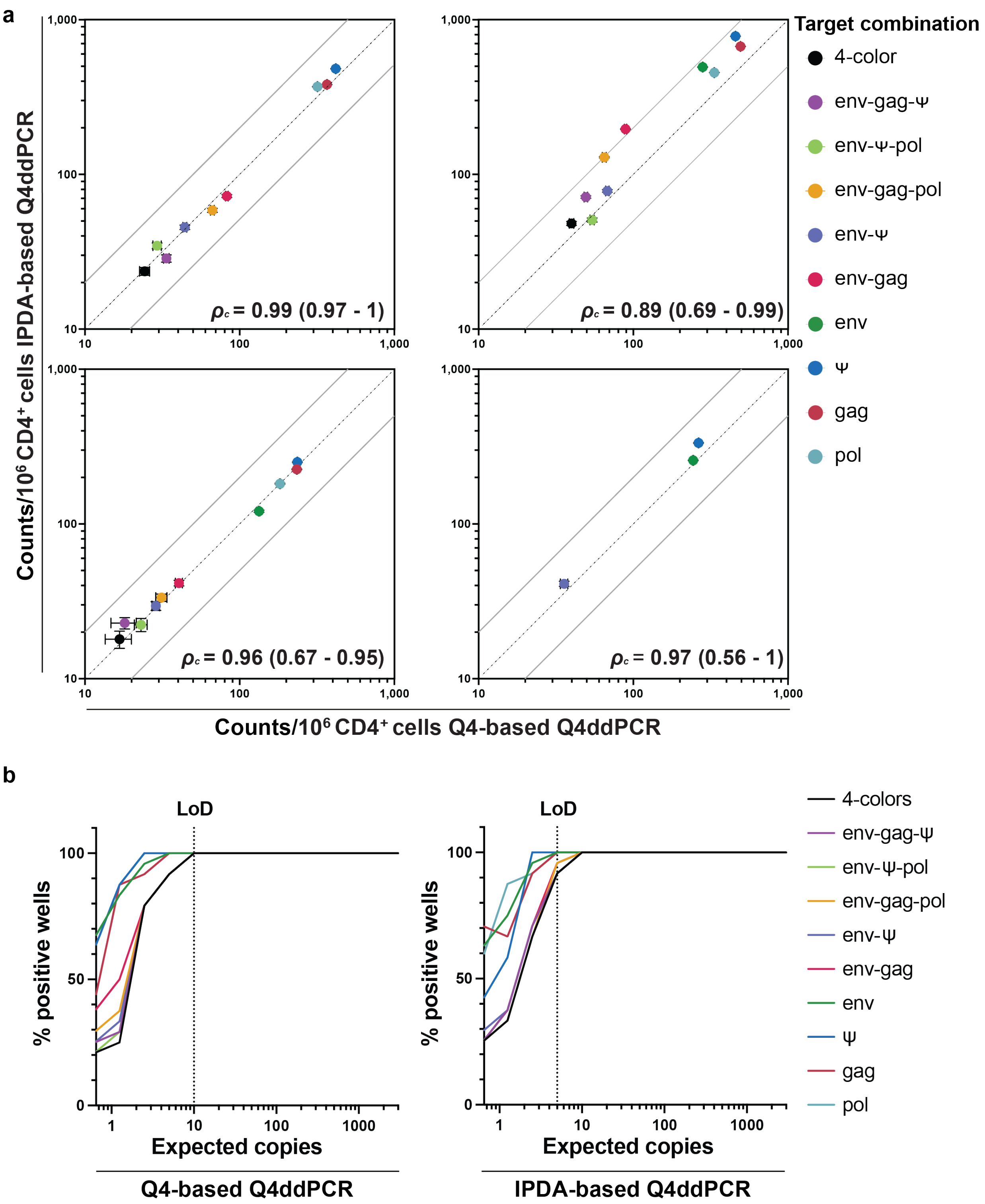
a
Counts/10^6 CD4+ cells IPDA-based Q4ddPCR
Counts/10^6 CD4+ cells Q4-based Q4ddPCR
ρc = 0.99 (0.97 - 1)
ρc = 0.89 (0.69 - 0.99)
ρc = 0.96 (0.67 - 0.95)
ρc = 0.97 (0.56 - 1)
Target combination
4-color
env-gag-Ψ
env-Ψ-pol
env-gag-pol
env-Ψ
env-gag
env
Ψ
gag
pol
b
LoD
% positive wells
Expected copies
Q4-based Q4ddPCR
IPDA-based Q4ddPCR
4-colors
env-gag-Ψ
env-Ψ-pol
env-gag-pol
env-Ψ
env-gag
env
Ψ
gag
pol

Supplementary Figure 3 | Variability assessment and Limit of Detection (LoD) of Q4ddPCR

a To evaluate both intra-assay and inter-assay variability of Q4ddPCR, we repeated the entire workflow including $CD4^+$ T-cell isolation, DNA extraction and Q4ddPCR 7 - 13 times in four participants with lower reservoir sizes (5 - 43 intact proviruses/10^6 $CD4^+$ cells). For each participant, single-target and intact Q4ddPCR readouts (color-coded) were compared between assay variants. Each plot depicts one participant, geometric mean values and geometric standard deviations for Q4-based (x-axis) and IPDA-based (y-axis) Q4ddPCR are shown. Central dotted line indicates perfect agreement between assays; the flanking lines represent a 2-fold deviation. Concordance correlation coefficients (ρ_c) with 95% confidence intervals between Q4-based and IPDA-based measurements are shown.

b DNA from J-Lat 6.3 cells spiked into PBMCs from people without HIV (PWoH) was analyzed in three independent 10-step dilution series (0.625 - 3000 copies) to determine the LoD of Q4ddPCR. The LoD was defined as the lowest input copy number at which ≥95% of wells were positive. The percentage of positive wells is shown for each target combination (color-coded). Dotted lines indicate the LoD for Q4-based Q4ddPCR (10 copies) and IPDA-based Q4ddPCR (5 copies). Source data are provided as a Source Data file.

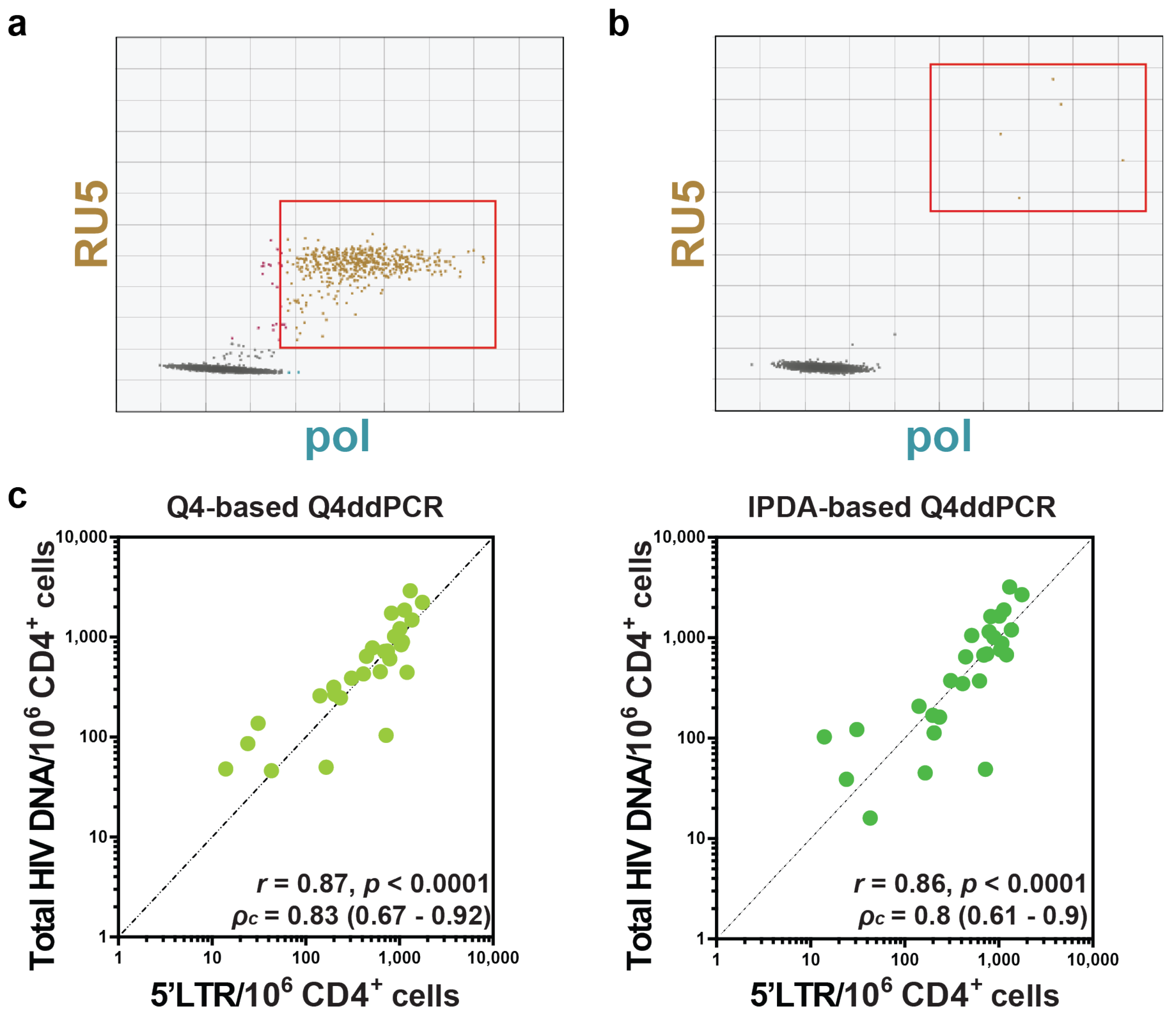
a
RU5
pol
b
RU5
pol
c
Q4-based Q4ddPCR
IPDA-based Q4ddPCR
Total HIV DNA/10^6 CD4^+ cells
10,000
1,000
100
10
1
r = 0.87, p < 0.0001
ρc = 0.83 (0.67 - 0.92)
r = 0.86, p < 0.0001
ρc = 0.8 (0.61 - 0.9)
5'LTR/10^6 CD4^+ cells

Supplementary Figure 4 | Comparison of total HIV DNA quantification by 5′LTR ddPCR and Q4ddPCR

a, b Single-probe ddPCR assay using the RU5 probe to quantify total HIV DNA by targeting the 5′LTR region, shown for J-Lat 6.3 DNA (a) and one participant sample (b). Signal from the ROX channel (RU5, y-axis) spills over into the ATTO590 channel (pol, x-axis). Red squares highlight falsely positive droplets for *pol*.

c Total HIV DNA measured by Q4ddPCR (y-axis) shows a strong correlation and high concordance with 5′LTR ddPCR (x-axis) in 28 people with HIV. Two-sided Spearman correlation (*r*- and *p*-values) and concordance correlation coefficients (ρ_c) with 95% confidence intervals are shown. Each dot represents one sample. Q4-based (light green) and IPDA-based (dark green) Q4ddPCR total HIV DNA are displayed. Source data are provided as a Source Data file.

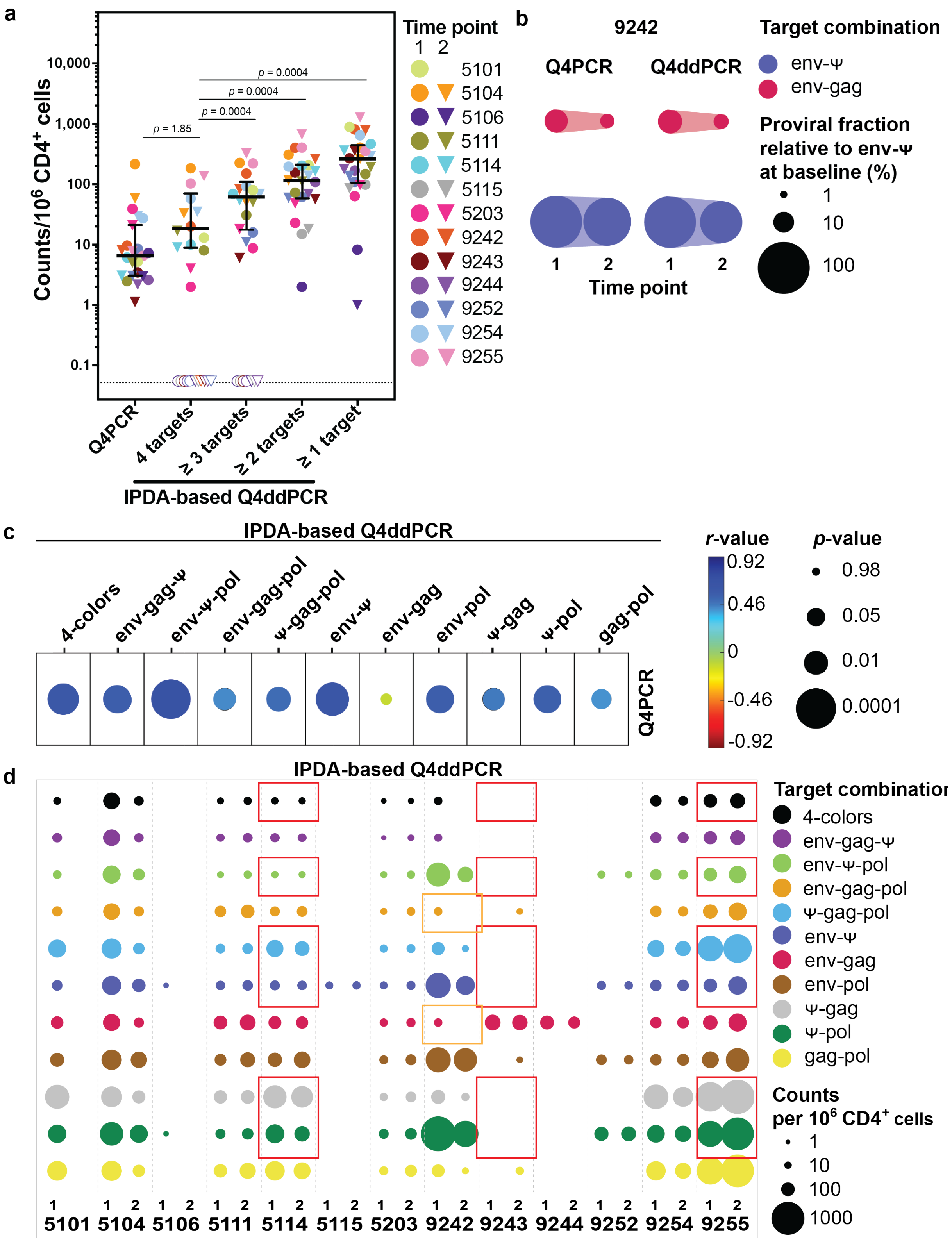
a
Counts/10^6 CD4+ cells
p = 1.85
p = 0.0004
p = 0.0004
p = 0.0004
Q4PCR
4 targets
≥ 3 targets
≥ 2 targets
≥ 1 target
IPDA-based Q4ddPCR
Time point
1 2
5101
5104
5106
5111
5114
5115
5203
9242
9243
9244
9252
9254
9255
b
9242
Q4PCR
Q4ddPCR
Time point
Target combination
env-Ψ
env-gag
Proviral fraction relative to env-Ψ at baseline (%)
c
IPDA-based Q4ddPCR
4-colors
env-gag-Ψ
env-Ψ-pol
env-gag-pol
Ψ-gag-pol
env-Ψ
env-gag
env-pol
Ψ-gag
Ψ-pol
gag-pol
Q4PCR
r-value
p-value
d
IPDA-based Q4ddPCR
Target combination
4-colors
env-gag-Ψ
env-Ψ-pol
env-gag-pol
Ψ-gag-pol
env-Ψ
env-gag
env-pol
Ψ-gag
Ψ-pol
gag-pol
Counts per 10^6 CD4+ cells
5101 5104 5106 5111 5114 5115 5203 9242 9243 9244 9252 9254 9255

Supplementary Figure 5 | Validation of Q4ddPCR on longitudinal samples from 13 people with HIV (PWH)

Q4ddPCR was applied to longitudinal samples from 13 PWH previously characterized using Q4PCR. Q4PCR combines a 4-target qPCR with near full-length genome sequencing and shares the *env, gag* and *pol*-primer/probe sequences with IPDA-based Q4ddPCR and all primer/probe sequences with Q4-based Q4ddPCR. All samples positive for ≥2 targets by Q4PCR had previously been sequenced, resulting in 3,650 proviral sequences for these 13 PWH.

a Proviral counts per 10^6 $CD4^+$ T cells measured by IPDA-based Q4ddPCR across different readout combinations, compared to intact proviral counts from Q4PCR. Cumulative counts from IPDA-based Q4ddPCR are grouped by the number of detected targets (1-4). Each symbol represents an individual sample (n = 25); circles indicate the first time point (n = 13), triangles the second (n = 12). Samples from the same participant are color-matched. Samples with zero detected proviruses for a given target number are plotted with transparent symbols on the dotted line. Medians and interquartile ranges are shown. Statistical comparisons used the two-sided Wilcoxon signed-rank test with post hoc correction for multiple comparisons.

b Dynamic of sequence-confirmed intact proviruses detected by Q4PCR (left) and corresponding Q4-based Q4ddPCR readouts (right) in participant 9242. Proviruses are stratified by target combinations including *env-Ψ* and *env-gag*. For direct comparison between Q4PCR and Q4ddPCR, proviral counts were normalized to *env-Ψ*-positive proviruses detected at the first time point for each assay. Q4ddPCR

captured the dynamics of both *env-Ψ-* and *env-gag*-positive sequence-confirmed intact proviruses. Circle size indicates the relative proviral fraction compared to *env-Ψ* population at the first time point; colors denotate target combination used to detect proviruses.

c Two-sided Spearman correlation between IPDA-based Q4ddPCR readouts and reservoir size as previously measured by Q4PCR. Correlations are shown for different Q4ddPCR target combinations (columns) with intact proviruses per 10^6 $CD4^+$ T cells measured by Q4PCR. Spearman's *r*-values are color-coded (blue to red); circle size indicates *p*-value.

d Frequency of proviruses positive for various 2-, 3-, or 4-target combinations per 10^6 $CD4^+$ T cells across two time points in 13 PWH. Each circle represents one readout; size indicates abundance of each target combination; color denotes the specific combination. Participant IDs are shown along the x-axis; samples from two time points are labeled (1, 2), except for participant 5101 (single time point available). Red squares highlight samples with *Ψ* detection failure in one Q4ddPCR variant that was rescued by the alternate version (Fig. 2). Orange squares highlight sequence-confirmed intact, but *env-Ψ*-negative proviruses from participant 9242 that declined over time. IPDA-based Q4ddPCR results are shown. Source data are provided as a Source Data file.

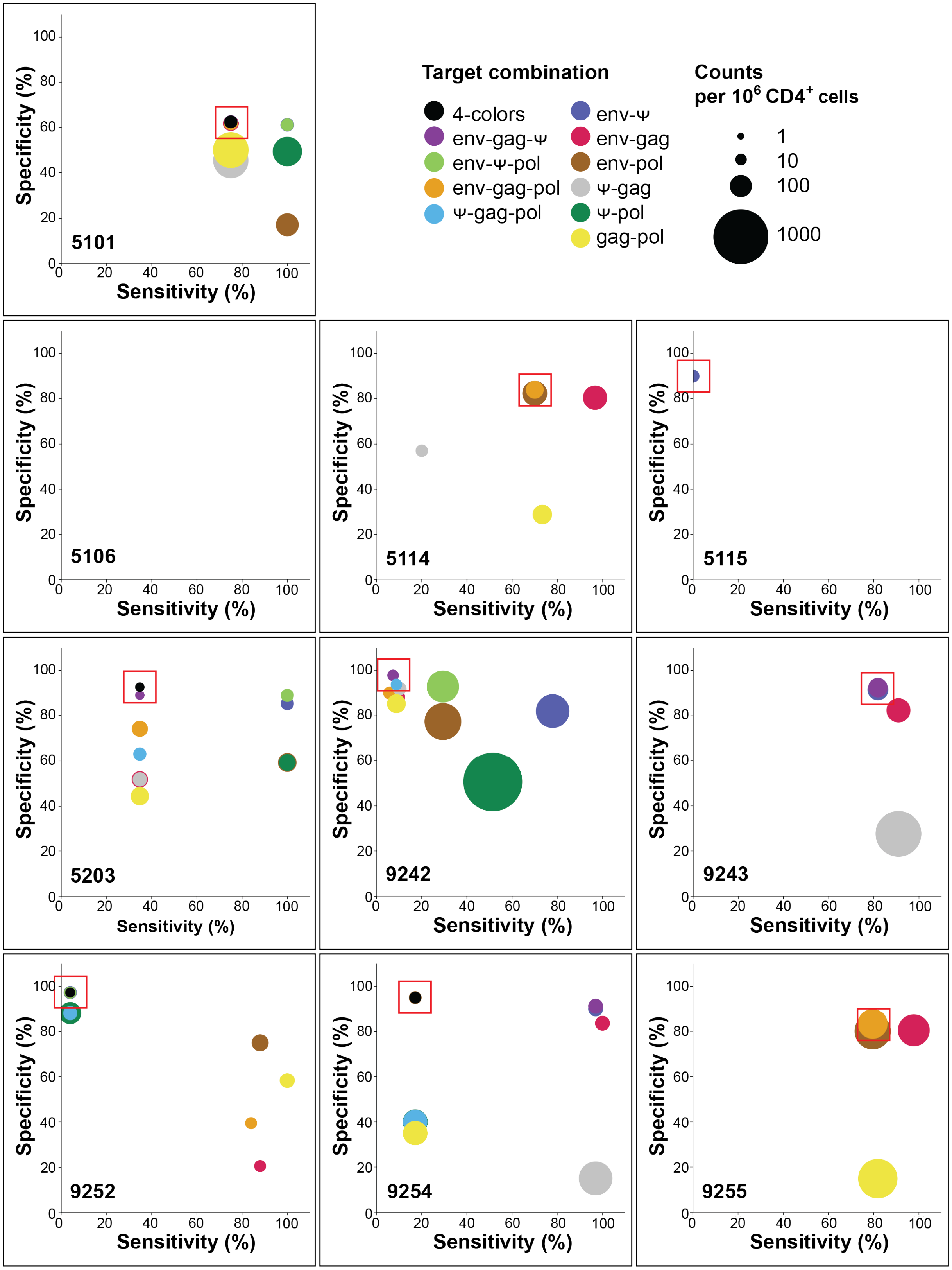

Target combination
4-colors
env-gag-Ψ
env-Ψ-pol
env-gag-pol
Ψ-gag-pol
env-Ψ
env-gag
env-pol
Ψ-gag
Ψ-pol
gag-pol
Counts per 10^6 $CD4^+$ cells
1
10
100
1000
Specificity (%)
Sensitivity (%)
5101
5106
5114
5115
5203
9242
9243
9252
9254
9255

Supplementary Figure 6 | Sensitivity and specificity of Q4ddPCR readouts for detecting intact HIV proviruses.

Sensitivity and specificity for intact provirus detection were evaluated across distinct Q4ddPCR target combinations using participant-matched Q4PCR-derived near full-length proviral sequences as a reference across 13 people with HIV. Sensitivity was calculated as the number of sequence-confirmed intact proviruses detected by a given target combination divided by the total number of intact sequences. Specificity was defined as the fraction of defective sequences not detected by the same target combination, relative to all defective sequences. Primer and probe sequences of Q4-based Q4ddPCR match those from Q4PCR. Panels show data from Q4-based Q4ddPCR results of individual participants. Circles denote specific target combinations; size corresponds to the number of proviruses detected per 10^6 $CD4^+$ T cells, and color encodes specific combination of amplified targets. Red squares mark the target combination selected by the decision tree. Source data are provided as a Source Data file.

Supplementary Tables

Supplementary Table 1 | Q4ddPCR amplicon locations and lengths

Target	For Primer	Rev Primer	Probe	Unlabeled probe	Amplicon length (bp)
**env**	7736 → 7759	7832 ← 7851	7781 → 7796	7781 → 7798	96
**Q4-Ψ**	682 → 699	761 ← 780	736 ← 757	-	79
**IPDA-Ψ**	692 → 711	775 ← 797	740 ← 758	-	83
**gag**	1300 → 1323	1359 ← 1377	1325 → 1354	-	59
**pol**	2536 → 2562	2634 ← 2662	2586 → 2604	-	98
	**HXB2**				

Supplementary Table 1 | Q4ddPCR amplicon locations and lengths

Genomic positions and amplicon lengths of Q4ddPCR primers and probes mapped to the HIV-1 HXB2 reference genome.

Supplementary Table 2 | Reproducibility of Q4ddPCR readouts and targets

Q4-based	4-colors				env-gag-ψ				env-ψ-pol			
Expected copies	Average	CV%	LL95 CV%	UL95 CV%	Average	CV%	LL95 CV%	UL95 CV%	Average	CV%	LL95 CV%	UL95 CV%
**3000**	2692.29	7.19	4.95	9.44	2853.82	7.18	4.93	9.42	2699.91	7.18	4.94	9.43
**600**	515.66	10.36	7.2	13.52	537.52	10.33	7.18	13.48	516.68	10.38	7.21	13.55
**300**	264.21	11.41	7.93	14.89	279.23	10.22	7.11	13.34	264.69	11.37	7.9	14.84
**60**	49.09	26.73	18.57	34.88	53.06	22.73	15.8	29.66	49.18	26.62	18.5	34.74
**30**	25.75	27.83	18.65	37.01	27.44	25.1	16.82	33.37	25.75	27.83	18.65	37.01
**10**	10.34	48.69	33.47	63.92	10.77	46.25	31.79	60.72	10.34	48.69	33.47	63.92
**5**	4.87	59.16	41.11	77.2	5.17	60.45	42.01	78.88	4.87	59.16	41.11	77.2
**2.5**	2.91	81.65	56.12	107.18	2.91	81.65	56.12	107.18	2.91	81.65	56.12	107.18
**1.25**	0.81	191.12	132.82	249.41	0.91	172.84	120.12	225.55	0.9	173.61	120.66	226.56
**0.625**	0.58	214.33	148.96	279.71	0.67	190.35	132.29	248.41	0.58	214.33	148.96	279.71

Q4-based	env-gag-pol				env-ψ				env-gag			
Expected copies	Average	CV%	LL95 CV%	UL95 CV%	Average	CV%	LL95 CV%	UL95 CV%	Average	CV%	LL95 CV%	UL95 CV%
**3000**	3017.99	6.16	4.23	8.09	2909.13	7.19	4.94	9.44	3437.61	5.38	3.7	7.06
**600**	589.32	9.8	6.81	12.79	544.05	10.29	7.15	13.43	641.63	9.55	6.64	12.47
**300**	288.76	10.35	7.2	13.51	282.29	10	6.95	13.06	313.96	9.08	6.31	11.85
**60**	53.72	25.9	18	33.81	53.87	22.23	15.45	29.02	58.92	22.63	15.73	29.54
**30**	28.24	29.7	19.91	39.5	27.88	25.4	17.02	33.77	30.3	25.74	17.25	34.22
**10**	11.06	47.79	32.85	62.73	10.77	46.25	31.79	60.72	11.6	44.15	30.35	57.95
**5**	5.45	59.65	41.46	77.85	5.17	60.45	42.01	78.88	5.85	59.71	41.5	77.93
**2.5**	2.91	81.65	56.12	107.18	2.91	81.65	56.12	107.18	2.91	81.65	56.12	107.18
**1.25**	1.1	144.23	100.24	188.22	0.99	158.08	109.87	206.3	1.38	113.26	78.71	147.8
**0.625**	0.76	171.15	118.95	223.35	0.67	190.35	132.29	248.41	0.95	141.56	98.38	184.73

IPDA-based	4-colors				env-gag-ψ				env-ψ-pol			
**Expected copies**	**Average**	**CV%**	**LL95 CV%**	**UL95 CV%**	**Average**	**CV%**	**LL95 CV%**	**UL95 CV%**	**Average**	**CV%**	**LL95 CV%**	**UL95 CV%**
**3000**	2913.97	4.21	2.93	5.5	3206.72	4.67	3.25	6.1	2928.5	4.28	2.98	5.59
**600**	556.75	8.8	5.98	11.62	604.35	8.08	5.49	10.67	558.02	8.81	5.98	11.64
**300**	289.97	9.07	6.3	11.83	309.27	8.22	5.71	10.73	290.56	9.08	6.31	11.86
**60**	51.76	21.14	14.69	27.58	54.04	21.12	14.68	27.57	52.07	21.45	14.91	27.99
**30**	29.58	29.4	19.97	38.83	31.03	32.25	21.9	42.6	29.58	29.4	19.96	38.83
**10**	11.49	51.83	36.02	67.63	12.37	53.57	37.23	69.91	11.49	51.83	36.02	67.63
**5**	5.07	52.71	36.23	69.2	5.09	53.93	37.07	70.79	4.89	55.54	38.17	72.9
**2.5**	1.75	87.54	60.84	114.24	1.95	81.93	56.94	106.92	1.75	87.54	60.84	114.24
**1.25**	1.17	157.53	109.48	205.58	1.26	145.19	100.9	189.47	1.17	157.53	109.48	205.58
**0.625**	0.73	189.69	131.83	247.55	0.73	189.69	131.83	247.55	0.73	189.69	131.83	247.55

IPDA-based	env-gag-pol				env-ψ				env-gag			
**Expected copies**	**Average**	**CV%**	**LL95 CV%**	**UL95 CV%**	**Average**	**CV%**	**LL95 CV%**	**UL95 CV%**	**Average**	**CV%**	**LL95 CV%**	**UL95 CV%**
**3000**	3051.44	4	2.78	5.22	3297.74	4.82	3.35	6.29	3380.44	4.41	3.07	5.76
**600**	583.28	8.44	5.73	11.15	615.94	8.17	5.55	10.8	633.75	7.76	5.27	10.25
**300**	302.39	8.73	6.07	11.39	313.9	8.04	5.59	10.5	322.7	7.94	5.52	10.36
**60**	54.27	20.11	13.97	26.24	55.04	21.15	14.7	27.6	56.66	20.74	14.41	27.07
**30**	30.89	27.44	18.63	36.24	31.47	31.69	21.52	41.85	32.44	29.84	20.26	39.41
**10**	12.46	49.93	34.7	65.16	12.56	53.66	37.3	70.03	13.34	51.51	35.8	67.22
**5**	5.49	49.47	34.01	64.94	5.29	52.93	36.38	69.48	5.5	50.55	34.74	66.35
**2.5**	1.75	87.54	60.84	114.24	2.06	83.56	58.07	109.05	1.95	81.93	56.94	106.92
**1.25**	1.26	154.98	107.71	202.25	1.36	151.96	105.61	198.3	1.36	143.34	99.62	187.05
**0.625**	0.73	189.69	131.83	247.55	0.83	170.93	118.79	223.06	0.73	189.69	131.83	247.55

Supplementary Table 2 | Reproducibility of Q4ddPCR readouts and targets

Mean detected copies and corresponding coefficients of variation (CV%) with 95% confidence intervals for Q4-based and IPDA-based Q4ddPCR, measured in J-Lat 6.3 cells spiked into PBMCs from people without HIV. Combined results of three independent dilution curves each with eight replicates. Source data are provided as a Source Data file.

Supplementary Table 3 | Cohort Characteristics

	5'LTR measurement	QVOA
**Number of participants**	28 (1 female, 26 male, 1 unknown)	16 (all male)
**Age at sampling (years)**	55 (32 - 70)	57 (32 - 70)
**Time on ART (years)**	21 (13 - 36)	20 (13 - 32)

Supplementary Table 3 | Cohort Characteristics

Cohort characteristics of PWH whose samples were used to detect total HIV DNA by 5’LTR-ddPCR or QVOA. For two participants no information for time on ART was available. Medians and ranges are indicated.

Supplementary Table 4 | Cohort Characteristics

**Number of participants**	13 (1 female, 12 male)
**Age at sampling (years)**	43 (29 - 59)
**Time on ART (years)**	7 (3 - 21)

Supplementary Table 4 | Cohort Characteristics

All participants were enrolled in studies on broadly neutralizing antibodies and had undetectable viral load at sampling. While all were on suppressive ART at the first sampling time point, treatment was interrupted at the second time point as part of an analytical treatment interruption. Medians and ranges are indicated.

Supplementary Table 5 | Sensitivity and Specificity of Specific Target Combinations

Q4PCR Target Combination	Sensitivity (%)	Specificity (%)	Positive Predictive Value (%)	Negative Predictive Value (%)
≥ 1 target	82.3	41.8	20.3	96.4
≥ 2 targets	67.3	69.7	28.6	93.7
≥ 3 targets	55	86.6	42.5	91.7
4-targets	45.5	95.3	63.5	90.3
env	93.2	45.3	23.5	98.5
Ψ	79.2	63.9	28.4	95.8
gag	87.3	26.7	17.7	97.3
pol	69.4	31.3	15.4	94.1
env-Ψ	73.3	91.1	59.7	94.7
env-gag	81.7	63.7	28.9	96.3
env-pol	64.9	65.5	25.3	93.3
Ψ-gag	64.3	75	31.7	93.2
Ψ-pol	57	77.6	31.4	92
gag-pol	62.4	45.4	17.1	92.9
env-gag-Ψ	61.8	94.5	66.9	92.8
env-Ψ-pol	51.3	94.1	61.1	91.2
env-gag-pol	59.1	72	27.6	92.4
Ψ-gag-pol	47.8	85.6	37.5	90.7
4-color	45.5	95.3	63.5	90.3

Supplementary Table 5
