# Supplementary material for: Q4ddPCR: a flexible, 4-target assay for high-resolution HIV reservoir profiling
Source: Nat Commun. 2026 Feb 20;17:2975. doi: 10.1038/s41467-026-69413-0 (PMC13035820; doi:10.1038/s41467-026-69413-0)
Supplement: Supplementary file 3 — Reporting Summary [file 41467_2026_69413_MOESM3_ESM.pdf]

nature portfolio

Corresponding author(s): Christian Gaebler, R. Brad Jones

Last updated by author(s): 01/20/2026

Reporting Summary

Nature Portfolio wishes to improve the reproducibility of the work that we publish. This form provides structure for consistency and transparency in reporting. For further information on Nature Portfolio policies, see our Editorial Policies and the Editorial Policy Checklist.

Statistics

For all statistical analyses, confirm that the following items are present in the figure legend, table legend, main text, or Methods section.

n/a	Confirmed	
☐	☒	The exact sample size (*n*) for each experimental group/condition, given as a discrete number and unit of measurement
☐	☒	A statement on whether measurements were taken from distinct samples or whether the same sample was measured repeatedly
☐	☒	The statistical test(s) used AND whether they are one- or two-sided<br>*Only common tests should be described solely by name; describe more complex techniques in the Methods section.*
☐	☒	A description of all covariates tested
☐	☒	A description of any assumptions or corrections, such as tests of normality and adjustment for multiple comparisons
☐	☒	A full description of the statistical parameters including central tendency (e.g. means) or other basic estimates (e.g. regression coefficient) AND variation (e.g. standard deviation) or associated estimates of uncertainty (e.g. confidence intervals)
☐	☒	For null hypothesis testing, the test statistic (e.g. *F*, *t*, *r*) with confidence intervals, effect sizes, degrees of freedom and *P* value noted<br>*Give P values as exact values whenever suitable.*
☒	☐	For Bayesian analysis, information on the choice of priors and Markov chain Monte Carlo settings
☐	☒	For hierarchical and complex designs, identification of the appropriate level for tests and full reporting of outcomes
☐	☒	Estimates of effect sizes (e.g. Cohen's *d*, Pearson's *r*), indicating how they were calculated

Our web collection on statistics for biologists contains articles on many of the points above.

Software and code

Policy information about availability of computer code

Data collection	Q4ddPCR and IPDA data was collected using QX Manager Software, Standard Edition v2.0
Data analysis	GraphPad Prism Version 10.5.0<br>R 4.5.0<br>SnapGene Version 8.0.3<br>Adobe Illustrator (version 29.6.1)<br>Biorender (https://www.biorender.com/)<br>Q4ddPCR and IPDA data was first analyzed with QX Manager Software, Standard Edition v2.0<br>Custom codes were used for calculation of reservoir data and log-linear mixed effects model for reservoir decline. They have been archived under: https://doi.org/10.5281/zenodo.15791354 and https://doi.org/10.5281/zenodo.16414846

For manuscripts utilizing custom algorithms or software that are central to the research but not yet described in published literature, software must be made available to editors and reviewers. We strongly encourage code deposition in a community repository (e.g. GitHub). See the Nature Portfolio guidelines for submitting code & software for further information.

Data

Policy information about availability of data

All manuscripts must include a data availability statement. This statement should provide the following information, where applicable:

- Accession codes, unique identifiers, or web links for publicly available datasets
- A description of any restrictions on data availability
- For clinical datasets or third party data, please ensure that the statement adheres to our policy

Proviral sequence data has previously been published and is available in GenBank with the accession no. MN090187-MN090943, MT189273 - MT191008, MT191115 - MT191120, MW059111 - MW059266, MW059441 - MW059533, MW059602 - MW059688, MW060242 - MW063069. Source data are provided with this paper. Data involving human research participants are subject to the data protection constraints in the written informed consent signed by the study participants.
Code availability: Custom codes for calculation of reservoir data and log-linear mixed effects model for reservoir decline have been archived under: (https://doi.org/10.5281/zenodo.15791354 and https://doi.org/10.5281/zenodo.16414846.

Research involving human participants, their data, or biological material

Policy information about studies with human participants or human data. See also policy information about sex, gender (identity/presentation), and sexual orientation and race, ethnicity and racism.

Reporting on sex and gender

Of the 13 people with HIV (PWH) of whom we used samples to validate Q4ddPCR, no data on gender was available. Data on sex was self-reported, 1 person was female, 12 were male. In the cohort where we assessed total HIV DNA by 5'LTR ddPCR 1 person was female, 26 were male and for one participant no data on sex nor gender was available. All of the 16 PWH in the cohort were we performed QVOA were male sex and gender. For the KOHIVI cohort gender and sex matched for all participants and was self-reported: 9 female, 18 male. In the ACTG A5321 cohort we included 9 participants with female sex and gender and 33 with male sex. 32 reported male gender, for 1 participant no data on gender is available.

Reporting on race, ethnicity, or other socially relevant groupings

Among the 13 people with HIV (PWH) whose samples we used to validate the Q4ddPCR assay, ethnicity information was available for 4 individuals: 3 identified as Hispanic and 1 as non-Hispanic. Race was self-reported by 13 participants: 2 identified as American Indian, 5 as Black or African American, 5 as White, and 1 as multiracial. In the cohort where we assessed total HIV DNA by 5'LTR ddPCR race was reported as follows: 15 identified as white or Caucasian, 3 as Black or African American, 4 as Asian, 2 as East Asian, 1 as multiracial, 1 identified as Hispanic regardless of race. For 4 PWH there was no information on race. For the KOHIVI cohort, data on race and ethnicity were not available but data on country of birth (23/27 Germany, 2/27 Poland, 1/27 Slowakia, 1/27 Croatia). In the ACTG A5321 cohort, 13 participants identified as Hispanic (regardless of race), 1 as American Indian or Alaska Native, 6 as non-Hispanic Black or African American, and 22 as non-Hispanic White. In our analyses, we examined only HIV-1 subtype B. Due to the relatively small sample sizes, we did not stratify our analyses by race or ethnicity.

Population characteristics

Cohort characteristics are summarized in Tables 1 and 2 and Supplementary Tables 3 and 4.

Recruitment

Participants cohort were recruited by HIV care providers at Charité – Universitätsmedizin Berlin, Germany, Maple Leaf Medical Clinic Toronto, Canada, or the NewYork-Presbyterian Hospital at Weill Cornell Medicine, United States. For the Q4ddPCR validation cohort and the ACTG A5321 cohort, samples were obtained as part of a nested, retrospective study using previously collected and banked specimens. No new participant recruitment was conducted.

Ethics oversight

Ethical approval to conduct this study was obtained from University of Toronto, the Weill Cornell Medicine Institutional Review Boards and the Ethics Committee of Charité Universitätsmedizin Berlin (reference numbers EA2/077/23). The ACTG cohort was approved by several institutional review boards at the authors' institutions. All participants provided written informed consent for their participation in the study.

Note that full information on the approval of the study protocol must also be provided in the manuscript.

Field-specific reporting

Please select the one below that is the best fit for your research. If you are not sure, read the appropriate sections before making your selection.

[x] Life sciences [ ] Behavioural & social sciences [ ] Ecological, evolutionary & environmental sciences

For a reference copy of the document with all sections, see nature.com/documents/nr-reporting-summary-flat.pdf

Life sciences study design

All studies must disclose on these points even when the disclosure is negative.

Sample size

Samples from a total of 110 people with HIV (PWH) across four independent cohorts were included in this study. For benchmarking analyses, 3,650 near full-length proviral sequences from 13 individuals were used. Sample sizes were determined based on availability of clinical specimens.

Data exclusions

Q4ddPCR samples were excluded only if total droplet counts were below 7,500 or if fewer than 40,000 cell equivalents were measured. For IPDA analyses, samples with amplification failure at either target were excluded from statistical comparisons. Log-transformed analyses

	excluded no samples; values of zero were set to one for transformation purposes only. Identical proviral sequences detected multiple times were counted only once in the sequence-based analyses.
Replication	Q4ddPCR experiments were conducted in technical replicates of 4–20 wells per sample. Where sample availability permitted, experiments were independently repeated. All replication attempts yielded consistent results within expected limits of reproducibility.
Randomization	Randomisation was not applicable in this study, as different reservoir quantification methods were directly compared using the same biological samples.
Blinding	This was an observational study and there was no allocation to experimental groups.

Reporting for specific materials, systems and methods

We require information from authors about some types of materials, experimental systems and methods used in many studies. Here, indicate whether each material, system or method listed is relevant to your study. If you are not sure if a list item applies to your research, read the appropriate section before selecting a response.

Materials & experimental systems

n/a	Involved in the study
☒	☐ Antibodies
☐	☒ Eukaryotic cell lines
☒	☐ Palaeontology and archaeology
☒	☐ Animals and other organisms
☐	☒ Clinical data
☒	☐ Dual use research of concern
☒	☐ Plants

Methods

n/a	Involved in the study
☒	☐ ChIP-seq
☒	☐ Flow cytometry
☒	☐ MRI-based neuroimaging

Eukaryotic cell lines

Policy information about cell lines and Sex and Gender in Research

Cell line source(s)	J-Lat Full Length Cells (6.3), ARP-9846 and MOLT-4/CCR5 Cells (ARP-4984) were obtained through the NIH HIV Reagent Program, Division of AIDS, NIAID, NIH
Authentication	J-Lat and MOLT-4/CCR5 cells were obtained directly from the NIH AIDS Reagent Program, which authenticates and distributes HIV reagents (https://www.beiresources.org/HIV.aspx). HIV-1 to J-Lat cell ratio measured by Q4ddPCR was as expected 1. MOLT/CCR5 Cells were tested for CD4 and CCR5 expression by flow cytometry.
Mycoplasma contamination	J-Lat and MOLT-4/CCR5 cells were obtained directly from the NIH AIDS Reagent Program, which tested them negative for mycoplasma contamination.
Commonly misidentified lines (See ICLAC register)	NA

Clinical data

Policy information about clinical studies

All manuscripts should comply with the ICMJE guidelines for publication of clinical research and a completed CONSORT checklist must be included with all submissions.

Clinical trial registration	NA
Study protocol	This study is a nested, observational study that utilizes peripheral blood mononuclear cell (PBMC) samples collected either as part of previously published studies or from the KOHIVI cohort at Charité – Universitätsmedizin Berlin or observational study cohorts at the University of Toronto and Weill Cornell Medicine. No interventional procedures were performed as part of this specific study.
Data collection	Participants in the KOHIVI cohort whose samples were included in this study were recruited between September 2023 and December 2024. PBMCs were collected and cryopreserved as part of routine cohort follow-up and used for the Q4ddPCR and comparative analyses presented here.
Outcomes	The KOHIVI cohort is an observational study with exploratory endpoints focused on characterizing the HIV reservoir and host immune responses. The outcomes assessed in this study include the quantification and decay dynamics of genetically intact HIV proviruses as measured by Q4ddPCR in comparison with established methods (e.g., IPDA).

Plants

Seed stocks	NA
Novel plant genotypes	NA
Authentication	NA
